# Evaluatıon of the protective role of melatonın ın methanol ınduced optıc neuropathy

**DOI:** 10.1007/s10792-025-03539-w

**Published:** 2025-05-09

**Authors:** Şerife Gülhan Konuk, Süleyman Özdemir, Raşit Kılıç, Gamze Bektur, Alper Güneş, Ender Şener

**Affiliations:** 1 Faculty of Medicine, Department of Ophthalmology, Tokat Gaziosmanpaşa University, Tokat, Turkey; 2 Faculty of Medicine, Department of Pathology, Tokat Gaziosmanpaşa University, Tokat, Turkey

**Keywords:** Melatonin, Methanol, Optic neuropathy, Optic nerve, Rat

## Abstract

**Purpose:**

To investigate the effects of intraperitoneal melatonin administration on the optic nerve and retina following acute methanol exposure.

**Materials and methods:**

Twenty-four female albino Wistar rats weighing 200–300 g and aged between 3 and 6 months were utilized. The rats were divided into three groups, each consisting of eight rats: healthy control (C), methanol (M), and methanol + melatonin (MM) groups. Initially, rats in groups M and MM were administered intraperitoneal methotrexate at a dose of 0.3 mg/kg. One week later, the same groups were orally given methanol at a concentration of 20% and a dose of 3 g/kg to induce methanol toxicity. To ensure survival, four hours after oral methanol administration, ethanol was orally administered at a concentration of 20% and a dose of 0.5 g/kg. Additionally, starting from the next day, the MM group received intraperitoneal melatonin at a dose of 20 mg/kg/day for 14 days. On the 14th day, rats were sacrificed, and their eyes, including the optic nerves, were enucleated for histopathological examinations. Myelin basic protein (MBP), retinal ganglion cell (RGC), glial cell degeneration and optic nerve thickness were evaluated.

**Results:**

The experiment was completed with a total of twenty-four rats, with each group consisting of eight rats. When evaluating RGC, glial cell degeneration, and optic nerve thickness, the results for Group MM were significantly better than those for Group M (*p* < 0.0001, *p* < 0.0001, *p* < 0.0001, respectively). There was no significant difference between Group MM and Group C, which was not subjected to alcohol intoxication (*p*: 0.89, *p*: 0.82, *p*: 0.77, respectively). There was no significant difference in MBP values ​​between the groups (*p*: 0.44, *p*: 0.17, *p*: 0.80, respectively).

**Conclusion:**

Intraperitoneal administration of melatonin has a significant positive effect on the structure of the retina and optic nerve resulting from methanol exposure. Melatonin should be considered in future studies as a potential therapy for methanol-induced toxic optic neuropathy.

## Introduction

Methanol poisoning occurs particularly in developing countries as a result of illegal consumption, suicide, or accidental intake of methanol [1]. Poisoning, which has high morbidity and mortality rates, manifests symptoms such as nausea, vomiting, and abdominal pain within the first 4 h. Central nervous system involvement and visual symptoms begin approximately 12 h later [2, 3]. Methanol poisoning can cause optic nerve and retinal damage, leading to symptoms ranging from blurred vision to total blindness. Symptoms may include photophobia, diplopia, and color vision impairment. The examination, optic nerve edema, hyperemia, pallor, and abnormalities in pupil reflex may be observed. Optic nerve atrophy develops slowly after toxic substance intake and usually completes within 30–60 days [4].

Various antidotes such as ethanol are used for the treatment of methanol poisoning; however, there is currently no defined effective treatment method for toxic optic neuropathy caused by methanol [5, 6].

Melatonin, secreted from the pineal gland, is a neurohormone responsible for regulating circadian rhythm and possesses antioxidant, anti-inflammatory, immunomodulatory, and neuroprotective properties [7, 8]. It has been shown in various studies that melatonin is produced in accordance with the circadian rhythm in the eye and receptors are present in various tissues of the eye [9, 10]. Studies have also shown that melatonin provides neuroprotection in the eye and protects retinal ganglion cells [4, 11].

Based on this information, we researched the effects of melatonin on the optic nerve and retinal damage resulting from methanol. To our knowledge, this is the first study in the literature assessing the protective effects of melatonin in methanol-induced optic neuropathy.

## Method

This research adhered to the principles outlined in the NIH statement for the Use of Animals in Research. Ethical approval for the study was obtained from the Animal Experimentation Ethics Committee of Tokat Gaziosmanpaşa University (Approval No: 2022 HADYEK-09).

Twenty four female Wistar albino rats, aged between 3 and 6 months and weighing 200–300 g, were utilized for the study. The rats were housed under standard laboratory conditions, which included a 12-h light–dark cycle, ambient light, and a room temperature of 20–22 °C.

The rats were divided into three groups, each consisting of 8 rats: Groups C, M, and MM. Group C served as the healthy control group and received no agents. Group M received methanol, ethanol, and methotrexate, while Group MM received melatonin treatment in addition to the agents administered to Group M. Initially, rats in Groups M and MM were given intraperitoneal methotrexate at a dose of 0.3 mg/kg. One week later, the same groups were orally administered methanol at a concentration of 20% and a dose of 3 g/kg to induce toxicity. To ensure survival, 4 h after oral methanol administration, oral ethanol at a concentration of 20% and a dose of 0.5 g/kg was administered to all groups. Additionally, starting from the next day, Group MM received intraperitoneal melatonin at a dose of 20 mg/kg/day for 14 days.

Methotrexate, a folic acid antagonist, was administered to sensitize rats to methanol toxicity since rats are folate-rich species and have low sensitivity to methanol intoxication [12].

At the end of the procedure, the rats were injected with 50 mg/kg intraperitoneal (i.p.) ketamine hydrochloride and 7.5 mg/kg i.p. xylazine for anesthesia. Following the attainment of appropriate anesthetic depth, the right eyes of all twenty-four rats from each group were enucleated under anesthesia. After enucleation, a conjunctival peritomy was performed by circumferentially cutting the conjunctiva, followed by dissection of the optic nerve posteriorly after optic nerve transection. Following the procedure, the experimental animals were euthanized by cervical dislocation under ketamine and xylazine anesthesia.

## Histopathological examination

**Fig. 1:**
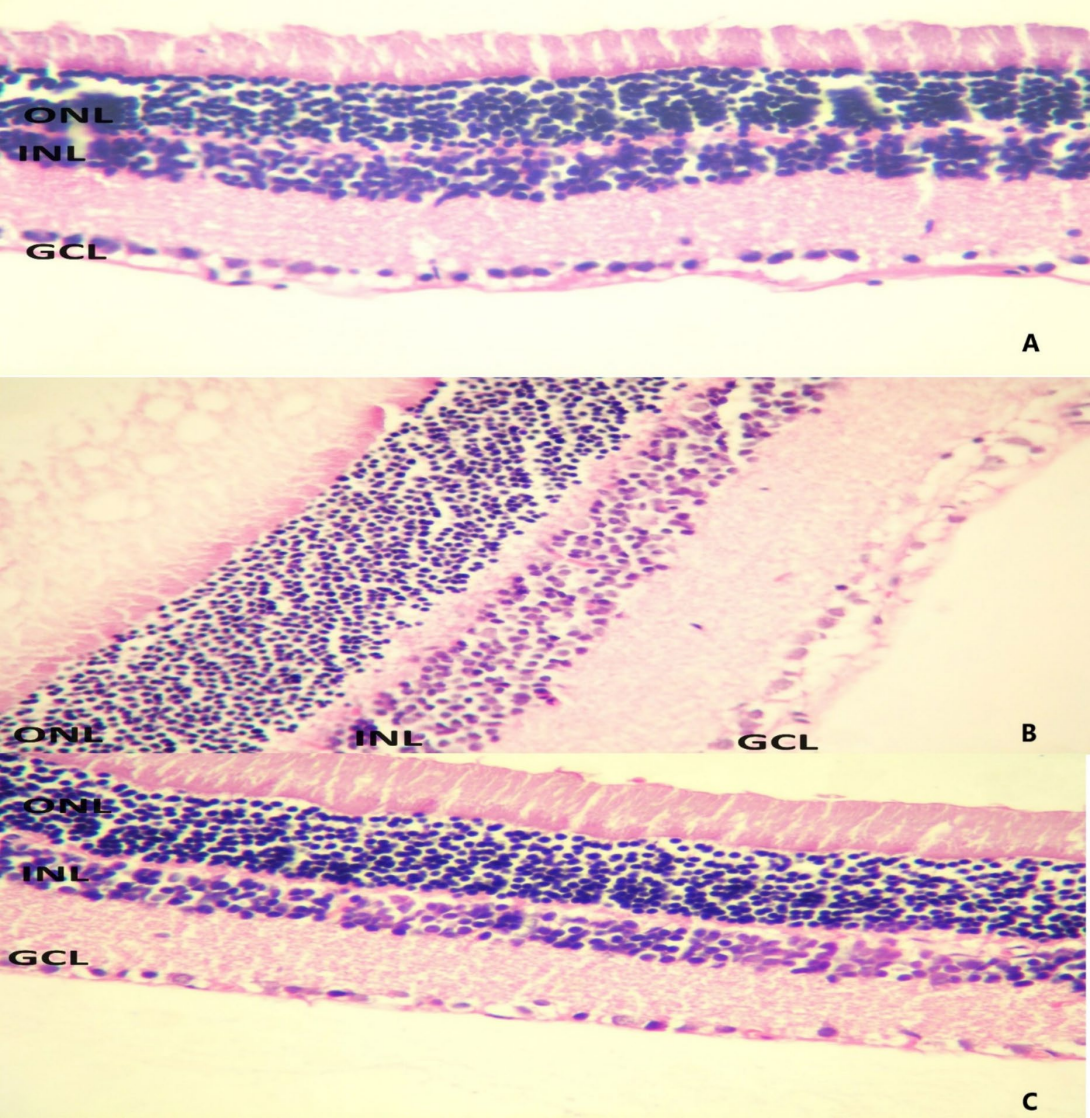
Retinal specimen **A** The control group specimen shows normal structure and no cellular swelling (score 5/5), **B** Group MM specimen shows severe structural anomaly, cellular swelling and vacuolation (score 1/5), **C** Group M specimen shows mild structural anomaly, cellular swelling and vacuolation (score 4/5). GCL (ganglion cell layer), INL (internal nuclear lamina), ONL (outer nuclear lamina), 400X

The orbital samples were embedded in paraffin after dissection. The obtained samples were stained with hematoxylin and eosin. The evaluation was performed by a pathologist under a light microscope (Nikon ECLIPSE E200). For retinal assessment, the scoring system described by El-Bakary et al. was utilized for evaluating the ganglion cell layer. In the semi-quantitative examination: a score of 5 was assigned for a regular appearance in ganglion cells, a score of 4 for irregular appearance and vacuolization in less than 50% of cells, a score of 3 for irregularity in 50–80% of cells and vacuolization in less than 20% of cells, a score of 2 for irregularity in more than 80% of cells, vacuolization in more than 50% of cells, and swelling in less than 50% of cells, and a score of 1 for irregularity in more than 80% of cells, vacuolization in more than 50% of cells, and swelling [13] (Fig. 1).

**Fig. 2:**
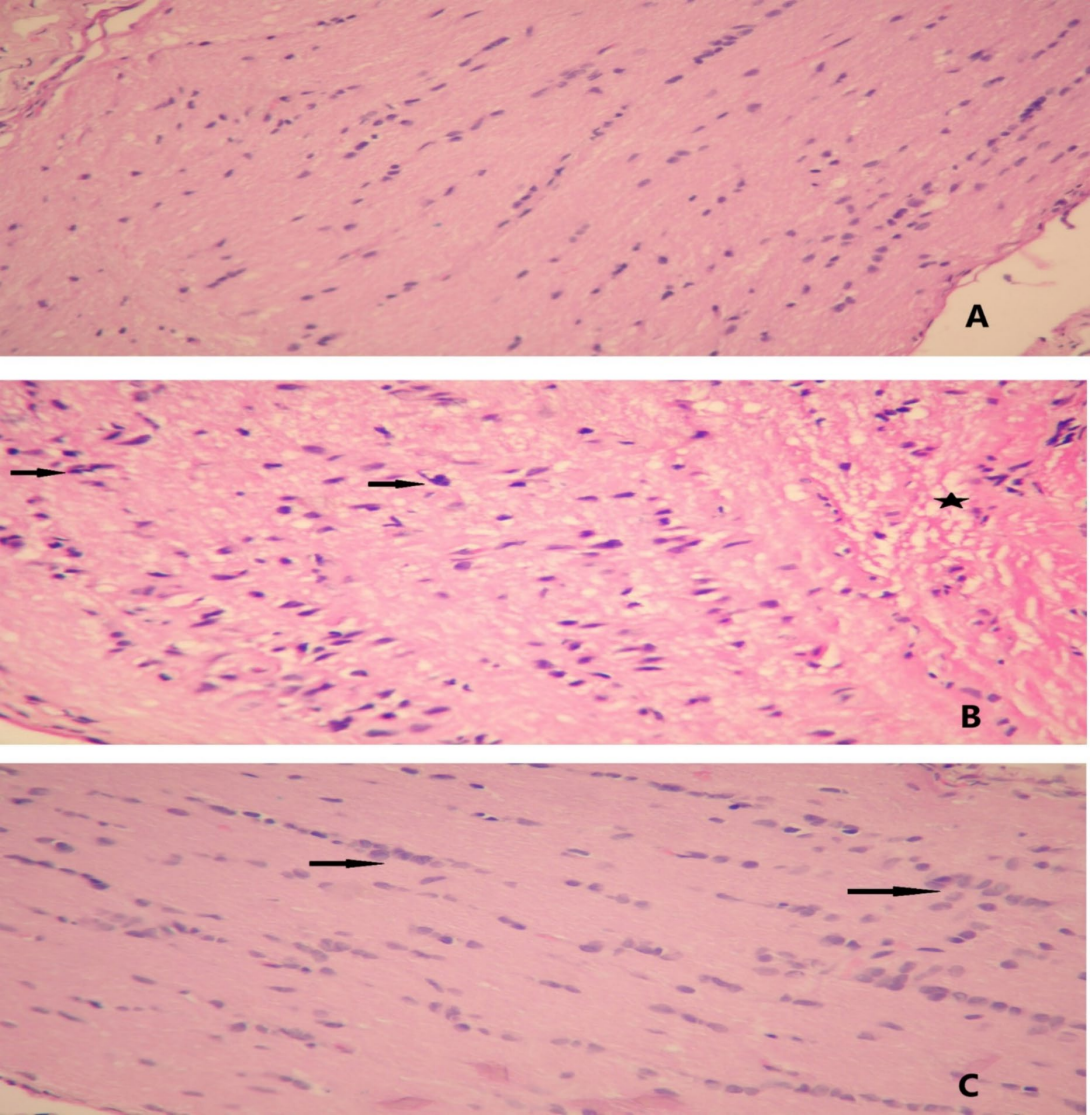
Optic nerve tissue. **A** Control specimen showing no sign of glial cell degeneration or thicken ing, (score 0), **B** Group 1 specimen shows severe glial cell degeneration (arrows) and vacuolization (star), (score 3), **C** Group 2 specimen shows minimal glial cell degeneration (arrows), (score 1), 400X

**Fig. 3:**
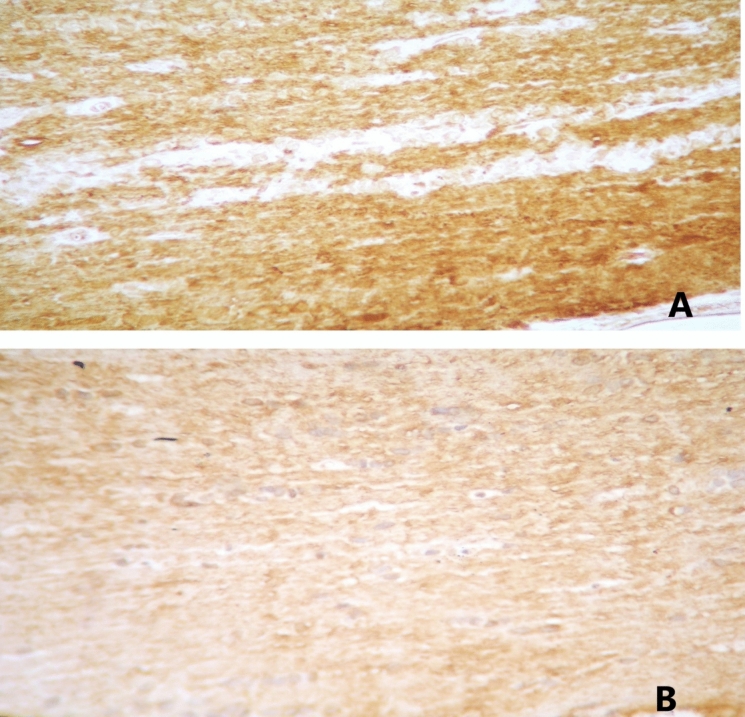
**A** Strong myelin basic protein expression on optic nerve tissue from control group specimen (score 3), 400X. **B** Moderate myelin basic protein expression on optic nerve tissue from group 1 specimen (score 2), 400x

In optic nerve samples, thickness increase and glial cell degeneration findings were evaluated semi-quantitatively. Each of these two parameters was evaluated separately as normal (score 0), mild degeneration or thickness increase (score 1), moderate degeneration or thickness increase (score 2), and severe degeneration or thickness increase (score 3) (Fig. 2). In the immunohistochemical study conducted to assess optic nerve damage, myelin basic protein (clone: YN01146m, Elabscience, USA) was applied to the samples using the Leica BOND-MAX device to demonstrate preserved myelin material. Staining intensity was evaluated as strong (score 3), moderate (score 2), and weak (score 1) (Fig. 3).

## Statistical analysis

Statistical analysis of the data was performed using SPSS (version 22.0; SPSS, Chicago, Illinois, USA). Numerical variables were summarized using mean ± standard deviation, median, and minimum–maximum values, while categorical variables were summarized using counts and percentages. The normality of the numerical data was assessed using the Kolmogorov–Smirnov test. One-way analysis of variance was used for comparisons among groups, followed by Tukey’s post hoc test. Statistical significance was set at *p* < 0.05.

## Result

A total of twenty four rats were enrolled in the study, and the study was completed without any loss. The results of the pathological evaluation are summarized in Table 1. There was no significant difference observed in MBP values among the groups. While there was no difference in optic nerve thickness between the C and MM groups, it was significantly thicker in Group M compared to both the C and MM groups. Furthermore, there was no difference in glial cell degeneration and RGC involvement in the optic nerve between the C and MM groups, whereas it was significantly better compared to Group M (Table 1).

**Table 1:** Results of pathological analysis

	Group C	Group M	Group MM	p1	p2	p3
MBP	3.00 ± 00	2.75 ± 0.46	2.62 ± 0.41	0.44	0.17	0.80
Optic nerve thickness	0.0 ± 0.0	1.62 ± 0.51	0.12 ± 0.35	0.00^*^	0.77	0.00^*^
Glial cell degeneration	0.0 ± 0.0	1.87 ± 0.64	0.12 ± 0.35	0.00^*^	0.82	0.00^*^
RGC	5.0 ± 0.0	2.25 ± 0.88	4.87 ± 0.35	0.00^*^	0.89	0.00^*^

C, control group; M, methanol group; MM, methanol-melatonin group; MPB, myelin basic protein; RGC, retinal ganglion cell

*Statistically significant; p1, significance level of the comparison between C and M groups; p2, significance level of the comparison between C and MM groups; p3, significance level of the comparison between M and MM groups

## Discussion

In our study, we investigated the protective and therapeutic effects of melatonin use on methanol induced optic neuropathy.

When examining the pathogenesis of alcohol poisoning, the alcohol dehydrogenase enzyme oxidizes methanol to formaldehyde, leading to the formation of formic acid. Since formic acid cannot be metabolized by humans, it causes toxic effects in the body. Formic acid binds to cytochrome C oxidase, located in the mitochondrial respiratory chain, resulting in cellular adenosine triphosphate (ATP) deficiency, electron transport issues, and a higher concentration of reactive oxygen species. This condition manifests as metabolic acidosis, end-organ failure, central nervous system depression, and vision loss. Visual impairments occur due to the direct effect of methanol or the oxidative damage caused by the toxic metabolite formic acid in retinal pigment epithelial cells and the optic nerve [14–16].

In cases of methanol poisoning, which have high mortality rates, an effective method has not yet been found. Among the treatment methods used for methanol poisoning, hemodialysis is applied to inhibit the hepatic metabolism of methanol, thus preventing metabolite formation and limiting metabolic acidosis [17]. Additionally, specific antidotes such as ethanol and fomepizole are administered to try to prevent the formation of formic acid, but these molecules are not effective against ocular toxicity. Intravenous methylprednisolone or erythropoietin applications are also being attempted for methanol induced optic neuropathy [18, 19].

Some experimental studies suggest that antioxidant molecules and neuroprotective agents may potentially be effective in the treatment of methanol induced optic neuropathy. İçel and al. [20] have argued that intravenous ATP administration in experimental methanol poisoning leads to significant improvements in the optic nerve and oxidative stress parameters. Taşlı and al. [21] have advocated for the effectiveness of Rutin usage in optic neuropathy due to its anti-inflammatory effects and its ability to reduce oxidative stress.

We utilized melatonin, a neurohormone secreted via the pineal gland, which is extensively researched for various aspects related to the eye, in our study [15]. Melatonin possesses numerous properties such as anti-inflammatory, antioxidant, immune modulating, and free radical scavenging capabilities [22]. Additionally, it exerts a stimulatory effect on neuronal branching, myelination, and maturation of oligodendrocytes, displaying protective effects on neurons [23]. The potent neuroprotective activity of melatonin arises from the combination of its anti-inflammatory and antioxidant effects.

In studies on oxidative stress parameters in alcohol poisoning, a significant decrease in total antioxidant values ​​was detected in the neural tissue of rats, and strong evidence was presented for the involvement of the inflammatory process [14, 21]. In this context, we examined the effects of melatonin on methanol poisoning-induced optic neuropathy in our study. Our findings support the positive effects of melatonin on the thickness of the optic nerve, glial cell degeneration, RBC oxidative damage and inflammation.

It is known that melatonin is considered a neuroprotective treatment in the eye due to its ability to reduce microglial activation. Increased microglial activation can lead to tissue damage and neuronal cell death by increasing the release of inflammatory cytokines [24]. Microglial activation is associated with retinal ganglion cell (RGC) death. Melatonin therapy may exhibit a neuroprotective effect by regulating microglial activation and inhibiting the release of proinflammatory cytokines [4, 11].

In an experimental study, melatonin therapy has been observed to increase the survival rate of RGCs and improve retinal function. This success has been attributed to the regulation of microglial activation and the inhibition of the TNFα-RGC p38 MAPK signaling pathway, thereby reducing apoptosis and necrosis of retinal cells [25].

In our study, significantly less susceptibility was detected in both RGCs and optic nerve glial cells, supporting the notion of melatonin’s microglial inhibition.

Our study has some limitations. One of them is the relatively low number of rats in the experimental study; however, we aimed to use the minimum number of animals to uphold animal rights. Another limitation is that the experimental conditions did not exactly replicate the clinical scenario. Nevertheless, the obtained results could serve as a basis for further investigation into the effects of melatonin therapy on methanol poisoning-induced optic neuropathy.

In conclusion, histopathological evaluations of the optic nerve support the promising effects of melatonin in the treatment of methanol-induced optic neuropathy. To the best of our knowledge, this study is the first to assess the role of melatonin in optic neuropathy in the literature. Future studies should include larger-scale and randomized controlled trials to better understand the effectiveness and safety of melatonin therapy.

## Data Availability

No datasets were generated or analysed during the current study.

## Declarations

### Confict of interest

The authors declare that they have no confict of interest.
